# Oncofetal HMGA2 effectively curbs unconstrained (+) and (−) DNA supercoiling

**DOI:** 10.1038/s41598-017-09104-5

**Published:** 2017-08-16

**Authors:** Xiaodan Zhao, Sabrina Peter, Peter Dröge, Jie Yan

**Affiliations:** 1 Mechanobiology Institute, National University of Singapore, 5A Engineering Drive 1, Singapore, 117411 Singapore; 2 School of Biological Sciences, Nanyang Technological University, 60 Nanyang Drive, SBS-02n-49 Singapore, 637551 Singapore; 3 Department of Physics, National University of Singapore 2 Science Drive 3, Singapore, 117542 Singapore; 4 Centre for Bioimaging Sciences, National University of Singapore, 14 Science Drive 4, Singapore, 117546 Singapore; 5 Graduate School for Integrative Sciences and Engineering, National University of Singapore, 28 Medical Drive, Singapore, 117456 Singapore

## Abstract

HMGA2 belongs to the family of the high mobility group (HMG) proteins. It binds DNA via three AT-hook domains to the minor groove of adenine-thymine (AT) rich DNA. Recently, a new function of HMGA2 as a replication fork chaperone that protects stem and cancer cells from replication fork collapse induced by chemotherapeutic agents was uncovered, suggesting a previously uncharacterized binding at replication forks. In this study, we examined HMGA2 binding to four DNA structures relevant to replication forks, namely ds DNA, ss DNA, forked DNA and supercoiled DNA plectonemes. We detected HMGA2 binding to supercoiled DNA at the lowest concentration and this binding mode transiently stabilizes the supercoiled plectonemes against relaxation by type I topoisomerase. Together, these findings suggest a plausible mechanism how fork regression and collapse are attenuated by HMGA2 during replication stress, i.e. through transient stabilization of positively supercoiled plectonemes in the parental duplex.

## Introduction

The oncofetal HMG AT-hook 2 (HMGA2) protein is expressed in humans during early development and plays crucial roles during proliferation and differentiation of cells^1–3^. It is upregulated in a variety of human cancers, although its precise role in cancer development is unclear^4, 5^. HMGA2 contributes to the regulation of gene expression and plays crucial roles globally in the formation of heterochromatic regions, such as telomeres and senescence-associated foci^6, 7^.

HMGA2 functions require interaction(s) with chromosomal DNA. The protein harbors three AT-hooks as unique DNA binding domains that preferentially recognize the minor groove of AT-rich duplex sequences^8, 9^. Therefore, DNA binding of HMGA2 is to some extent dependent on the sequence content of linear DNA. In addition, HMGA2 binding to short DNA fragments have shown that HMGA2 is a DNA bending protein that introduces a DNA bending angle of ~34°^10^.

Consensus high-affinity sequence motifs of 5’-ATATTCGCGAWWATT-3’ and 5′-ATATTGCGCAWWATT-3′ have been identified^9^. Compared to the many studies investigating the sequence-dependent binding of HMGA2, much less is known regarding its DNA structural feature dependent binding. A recent study has shown that HMGA2 also preferentially recognizes some specific structural features of DNA, such as holliday junctions and three-way junctions^11^. Preferential binding to such DNA structural features has been observed for the closely related HMGA1 protein, which also harbors three AT-hooks^12–14^.

**Figure 1:**
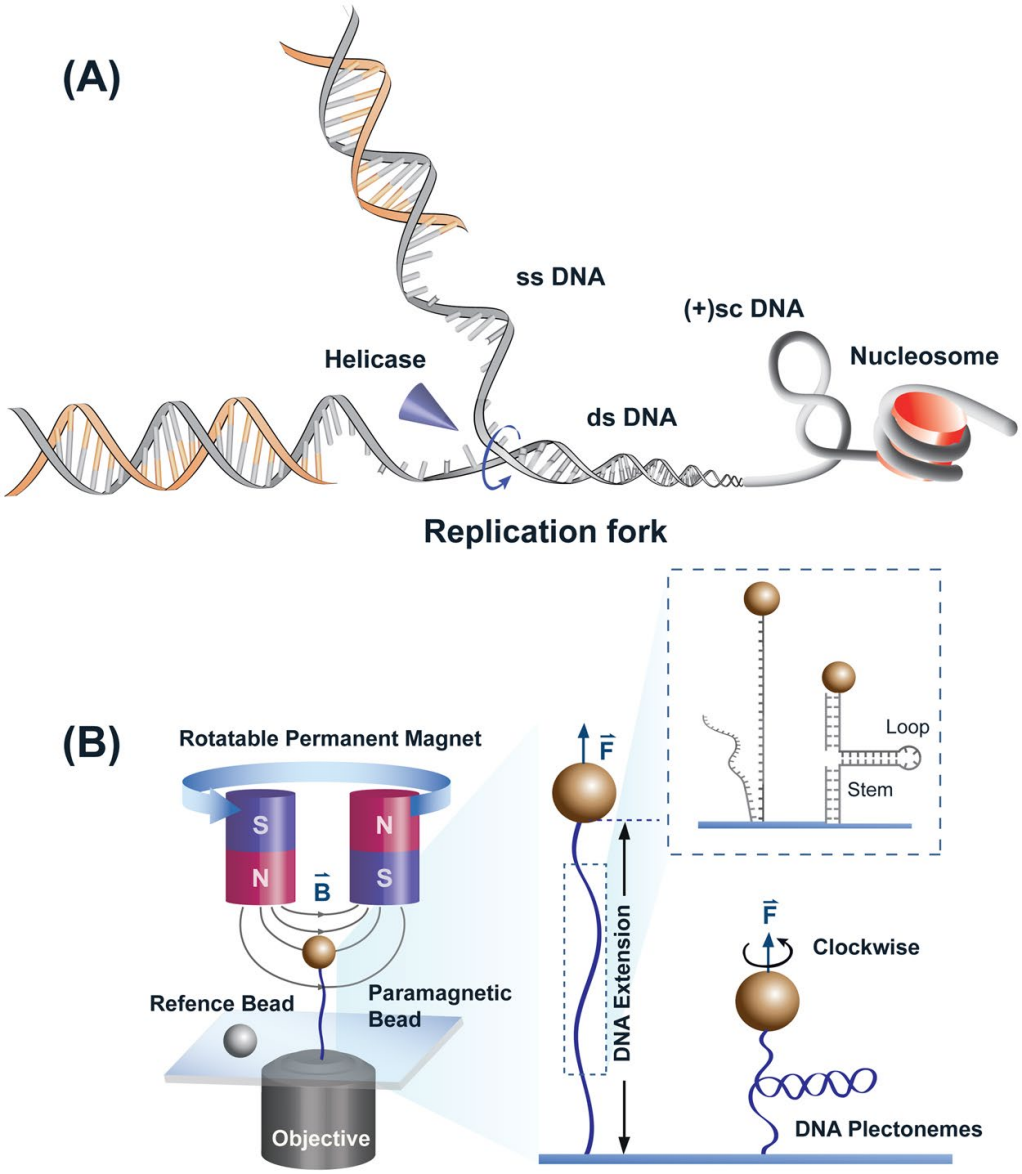
Schematics of DNA structures and magnetic tweezers. (**A**) Illustration of different DNA structures involved in replication fork. (**B**) Illustration of the magnetic tweezers setup. A single DNA, either torsionally unconstrained or constrained, is tethered between a glass coverslip and a paramagnetic bead. Force is applied to the molecule through the bead by an external magnetic field. For torsionally constrained DNA, the linking number is changed by rotating the magnets. Similarly, DNA constructs of ss DNA and forked DNA can be studied shown in the inset.

We have recently reported that HMGA2 protects stalled replication forks from collapsing into ds DNA breaks in *E. coli*, yeast and human model systems^11^. This suggested specific interaction(s) between HMGA2 and DNA structures at replication forks. Since replication forks can be stalled at random location, HMGA2 should not rely on specific DNA sequence contexts for higher affinity binding. Therefore, HMGA2 might recognize a specific DNA structure that is associated with replication and binds to it with high affinity that results in suppression of fork regression^11^. Several DNA structures are formed at a (stalled) replication fork, including ss DNA, a fork DNA junction and positively supercoiled plectonemes generated during fork progression in the parental DNA (schematics in Fig. 1A)^15, 16^. Besides ds DNA, binding of HMGA2 to three-way junctions and to ss DNA have been reported in previous bulk biochemical assays^11^.

In order to provide more insights into the mechanism by which HMGA2 protects stalled replication forks from collapsing into double-stranded breaks, we investigated the DNA mechanical responses altered under various concentrations of HMGA2 for different DNA constructs at a single DNA level using magnetic tweezers (schematics in Fig. 1B). We found that: (1) HMGA2 binding to ds DNA could be detected at ~500 nM and ss DNA at ~100 nM HMGA2 concentrations, (2) HMGA2 binding moderately stabilizes DNA fork from unfolding, (3) HMGA2 binding to supercoiled DNA (sc DNA) plectonemes or forked DNA could be detected in concentration range 20–50 nM, lower than that for linear dsDNA and ssDNA, and (4) binding of HMGA2 to supercoiled DNA strongly suppresses the relaxation of DNA plectonemes against topoisomerase treatment at higher concentrations (>200 nM). These results suggest at least one plausible mechanism for the observed DNA fork chaperone function of HMGA2, i.e. suppressing fork regression through a transient stabilization of the downstream positive supercoiled plectoneme at stalled replication fork. The potential implications of these findings for the protection of stalled replication forks by HMGA2 are discussed.

## Results

### HMGA2 compacts linear ds DNA molecules

**Figure 2:**
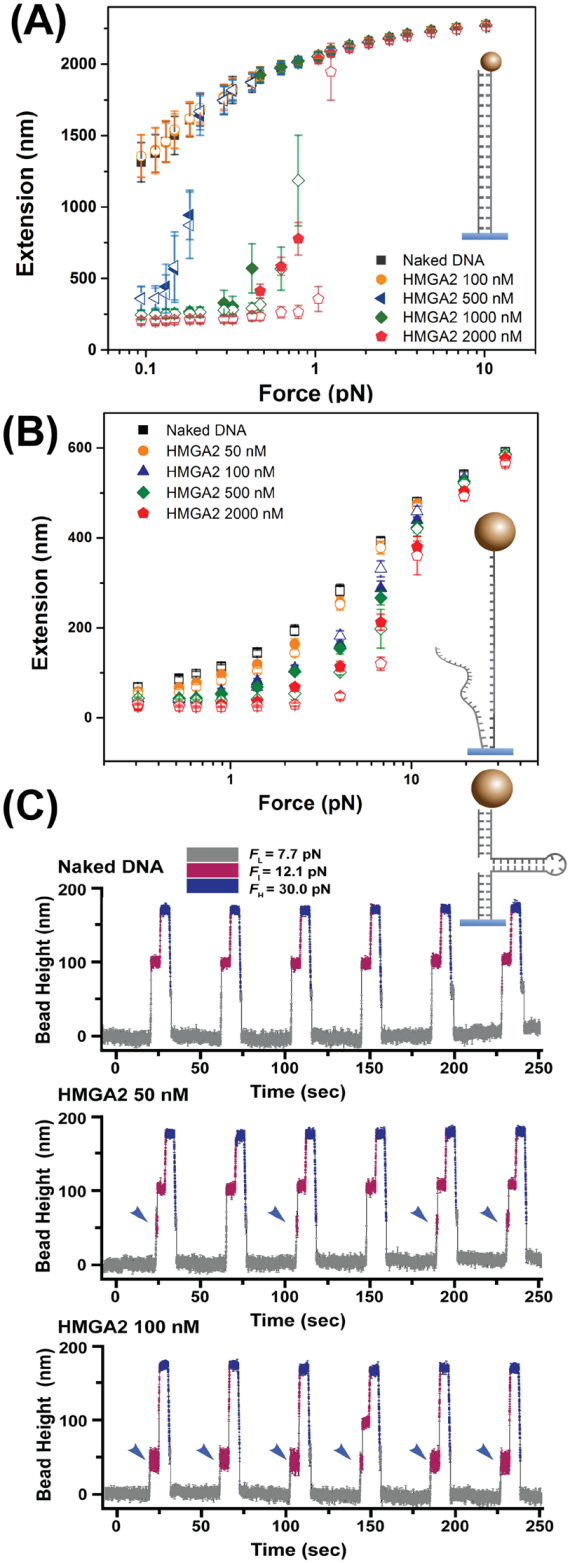
Binding of HMGA2 to ds DNA, ss DNA, and forked DNA. (**A**,**B**) Representative force-extension curves of a torsionally unconstrained ds DNA (**A**) and ss DNA (**B**) obtained in a force-decrease (solid data points) scan and a following force-increase scan (hollow data points) in different HMGA2 concentrations. Error bars are the standard deviation values of the DNA extension fluctuation over the recording time windows. (**C**) Representative time traces of bead heights of DNA fork during six force cycles at varied HMGA2 concentrations. The bead height at *F*_L_ is set as zero. In the absence of HMGA2 (top panel), after force jumping from *F*_L_ to *F*_I_, the bead height changed immediately to the level corresponding to that of unfolded hairpin (Fig. S4), indicating rapid hairpin unfolding at this force. In the presence of 50 nM (middle panel) or 100 nM HMGA2 (bottom panel), HMGA2 binding resulted in increased lifetime of the hairpin at *F*_I_ (marked by blue arrows) compared to the case in the absence of HMGA2 binding. In the measurements for (**A**,**B**), the change of DNA extension could be determined. In the measurement for (**C**), due to the significant influence of bead rotation during force change, accurate DNA extension change could not be determined. Therefore, the bead height change was used to present data (see details in Methods: “magnetic tweezers experiments”).

We first characterized how HMGA2 binds ds DNA in a single-molecule assay using magnetic tweezers (Fig. 1B). On the torsionally-unconstrained linear ds DNA, we obtained data of force-extension on the same DNA molecule at various HMGA2 concentrations, which were recorded during force-decrease and a subsequent force-increase scans (Fig. 2A; solid and hollow data points, respectively). At each force, the tether was held for 30 seconds to obtain the average extension. At 100 nM HMGA2, the force-decrease and force-increase curves overlapped, which coincided with those obtained before HMGA2 was introduced. This indicated that binding of HMGA2 to linear DNA was scarce and not sufficient to cause detectable conformational deformations of the DNA.

We then measured HMGA2 binding at 500 nM and found that at the extension is significantly shorter than with naked DNA when the force is below 0.2 pN, indicating DNA deformation by HMGA2 binding that caused a reduction in extension. The force-decrease and force-increase curves overlapped in most of the force range, i.e. without significant hysteresis, implying that the conformations of HMGA2-DNA complexes nearly reached a steady state over the force-changing time scale. Previous studies have shown that HMGA2 can introduce local DNA bends at its binding sites, with a DNA bending angle of ~34°^10^. Therefore, local DNA bending by AT-hooks is probably contributing to the reduction in extension^17^.

At higher HMGA2 concentrations ( > 1000 nM), the reduction in DNA extension occurred even at higher forces around 1 pN, with significant hysteresis between the force-decrease and force-increase curves **(**Fig. 2A **)**. Such hysteresis indicated that complicated HMGA2-DNA complexes likely formed other than simple local DNA bending. Extension fluctuations at constant forces revealed large amplitudes, indicating dynamic large-scale DNA condensation mediated by HMGA2 (Fig. S1). DNA condensation at high HMGA2 concentration was confirmed using atomic force microscopy (AFM) imaging (Fig. S2). These HMGA2 concentration-dependent effects on the force-extension curves of linear DNA were repeated and apparent DNA condensations were observed at HMGA2 concentrations greater than 300 nM (Fig. S3). Overall, these results suggest that HMGA2 binds to non-specific and linear ds DNA at > 300 nM concentrations, which involves DNA condensation at sufficiently high concentration.

### Binding of HMGA2 to ss DNA and forked DNA

We next tested ss DNA and forked DNA as substrates using magnetic tweezers. On ss DNA, we found that the protein is able to form complexes at around 100 nM (Fig. 2B). This binding is indicated by a moderate decrease in ss DNA extension in a few pN force range. Although the ss DNA binding activity is unexpected due to the lack of typical ss DNA binding domains such as OB fold, the interaction between ss DNA and HMGA2 was also suggested in a recent study of nucleic acid-protein interaction screen^18^. This ss DNA binding was observed at protein concentration greater than 50 nM, much higher than the *k*_D_ of the replication protein A (RPA) (~1 nM)^19^.

HMGA2 binding onto a DNA fork is shown in Fig. 2C. The mechanical stability of the DNA fork was characterized by the unzipping of the hairpin, indicated by a stepwise extension increase of ~50 nm at around ~12.1 pN. This hairpin remained stable at forces below 10 pN (Fig. S4). The HMGA2 binding to the junction was investigated in a force cycle procedure. In each cycle, the force applied to DNA was sequentially changed between three levels: a low level (*F*_L_ = 7.7 ± 0.8 pN) for 30 seconds to allow HMGA2 binding, an intermediate level (*F*_I_ = 12.1 ± 1.2 pN) for 5 seconds to detect whether the forked DNA is bound with HMGA2, and a high level (*F*_H_ = 30.0 ± 3.0 pN) for 5 seconds to remove any bound HMGA2. HMGA2 bound to the forked DNA is expected to increase the lifetime of the hairpin at *F*_I_ compared to the naked DNA, which can be detected in experiments.

Figure 2C top panel shows that in the absence of HMGA2, after jumping from *F*_L_ to *F*_I_, the height of the bead already reached the level corresponding to unfolded hairpin at *F*_I_ (Fig. S4). This indicates that the junction unfolded during the force-jump process before reaching *F*_I_ (note that it takes around 0.1 seconds for force jump to another level). In contrast, in the presence of 50 nM HMGA2, significantly increased the lifetime of the hairpin (>0.5 s) at *F*_I_ was observed in forty-three of fifty force cycles, indicating HMGA2 binding to the forked DNA during the time when the DNA was held at *F*_L_ in these cycles. At an increased concentration of HMGA2 (100 nM), HMGA2 binding was detected in all the fifty force cycles. In addition, in most of these cycles, the hairpin remained stable through the 5 seconds holding at *F*_I_. The lifetime histogram of the hairpin in the folded state at *F*_I_ was summarized in Fig. S5 with varied HMGA2 concentration. Overall, these results suggest that HMGA2 binding to the junction with a dissociation constant in the order of 50 nM and reached saturation at ~100 nM. The increased lifetime at 100 nM HMGA2 compared to that at 50 nM HMGA2 likely indicates that there is more than one HMGA2 bound to the DNA fork in the former case.

### Binding of HMGA2 to DNA plectonemes

We next tested HMGA2’s binding to supercoiled DNA plectonemes^15^. In our experimental set-up, both strands at the end of the DNA were tethered either to the coverslip surface or to the paramagnetic bead, such that the linking number (*Lk*) of the DNA can be changed by rotating the bead in a controllable manner^20^. Due to its double-helical conformation with 10.4 bp/helical turn^21^, *Lk* in topologically relaxed DNA of *N* bp equals *Lk*_0_ = *N*/10.4. The superhelical density $|\sigma |=(Lk-L{k}_{0})/L{k}_{0}$ of a DNA molecule, where *Lk* refers to the actual linking number of that molecule, is an important length-independent quantitative description of the deviation of the DNA linking number from the relaxed value. Since Δ*Lk* is (roughly) equal to the number of turns of bead rotations, the superhelical density can be calculated based on the number of bead rotations and the number of bp.

**Figure 3:**
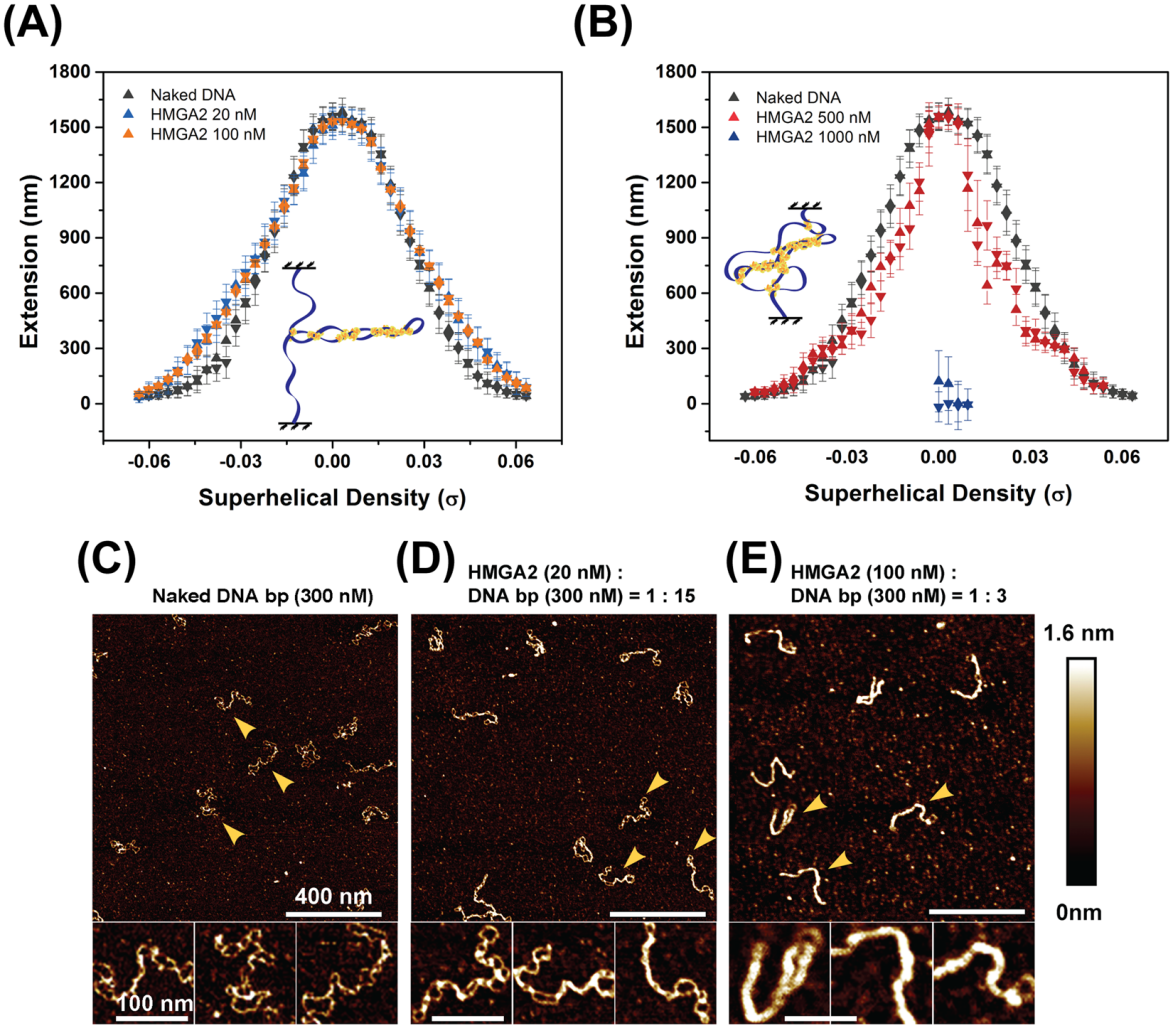
Binding of HMGA2 to sc DNA. (**A**,**B**) Representative *Lk*-extension curves of a torsionally constrained DNA at 0.3 pN in different HMGA2 concentrations. Data obtained in a forward process with increasing DNA winding ( + *σ*) or unwinding (−*σ*) are indicated by up-triangles, and those in a reverse process are indicated by down-triangles. The insets depict models of HMGA2-sc DNA complexes; see text for details. (**D**,**E**) AFM images of 2686 bp negatively supercoiled DNA bound with HMGA2 with different HMGA2: DNA base pair stoichiometric ratios. Images obtained on naked supercoiled DNA (**C**) are shown for comparison. In each panel in (**C**–**E**), three representative DNA molecules indicated by the arrows are enlarged three-fold for better visualization of the conformations of the supercoiled DNA molecules. The panel titles in (**C**–**E**) indicates the molar concentrations of HMGA2 and DNA basepairs as well as their ratios.

Changing *Lk* of an extended ds DNA causes DNA twist deformation and results in accumulation of twist elastic energy. After reaching a threshold, the accumulated energy is relaxed through chiral bending into plectonemic supercoiling. Formation of plectonemes, in turn, results in reduced DNA extension (Fig. 3A). As a tensile force has an effect against extension reduction, plectonemic supercoils form only at sufficiently low forces. Experimental and theoretical studies have shown that this occurs at forces below 0.5 pN^22–24^.

In the experiments, the supercoiling density was changed from one value to another by rotating the magnets with a speed of 6 rotations/second. At each value of supercoiling density, the DNA was held for 20 seconds during which the average extension of DNA was measured. Before HMGA2 was added to DNA, we observed a symmetric twist-extension curve, which is due to the formation of (+) or (−) supercoils under ~0.3 pN (Fig. 3A). The onset of (+/−) supercoiling is indicated by the superhelical density threshold values (${\sigma }_{\ast }\sim \pm 0.01$). At $|\sigma | > |{\sigma }_{\ast }|$, the DNA extension linearly decreases due to the formation of plectonemes. These data are in excellent agreement with previous experimental studies^20, 25, 26^ and theoretical predictions based on DNA bending and twisting stiffness^22, 27^.

In the presence of 20–100 nM HMGA2, the shape of the curves changed. The overall effect is that the extension of DNA increases in the supercoiling density range of $[0.03 < |\sigma | < 0.05]$, with a maximum of ~15% relative to naked DNA at $|\sigma | \sim 0.04$ (Fig. 3A; Fig. S6A). Such extended conformations of the (+) and (−) supercoiled molecule are likely due to the formation of HMGA2-DNA bridges inside the plectonemes, which reduces the size of the supercoiled loops and therefore releases additional DNA out into the extended region.

At 500 nM HMGA2, DNA extension in the relaxed state remained similar to that of naked DNA. However, unlike the ~15% extension increase in the supercoiling density range of $[0.03 < |\sigma | < 0.05]$ observed in 20–100 nM HMGA2, upon introduction of (+) or (−) DNA supercoiling $\,[0.01 < |\sigma | < 0.04]$, DNA extension was substantially reduced compared to naked DNA (Fig. 3B). Furthermore, investigation of the dynamics of extension fluctuation at constant supercoiling densities revealed large amplitudes of extension change, indicating transient DNA condensation (Fig. S6B). This effect became even more pronounced at 1000 nM HMGA2 where large-scale DNA condensation occurred at $|\sigma | \sim 0$ (Fig. 3B). Together, these data indicate that at > 500 nM, dynamic large-scale DNA condensation/decondensation occurred, which are similar to those observed on torsionally-unconstrained linear ds DNA at similar HMGA2 concentration.

Importantly, significant binding of HMGA2 to (+) and (−) sc DNA takes place at lower concentrations ( > 20 nM) than torsionally-unconstrained ds DNA ( > 300 nM), ss DNA ( > 50 nM) and forked DNA ( > 50 nM), indicating that sc DNA is the highest affinity binding substrate among the three DNA structures relevant to replication fork.

In order to obtain information on the conformations of the HMGA2-sc DNA complexes over this protein concentration range, we performed AFM imaging (Fig. 3C–E). The AFM images obtained with 2686 bp (−) supercoiled plasmids alone show expected braid-like conformations (Fig. 3C). In the presence of 20 nM HMGA2 (HMGA2:bp stoichiometric ratio of 1:15), significant changes in the DNA morphology were observed, with overall more extended braid conformation and increased apparent height (Fig. 3D), indicating HMGA2 binding. The binding was not saturated and HMGA2 formed continuous segmental tracts. Further increasing the concentration to 100 nM (HMGA2:bp stoichiometric ratio of 1:3) resulted in nearly complete coverage of sc DNA with HMGA2, and the generation of rod-like structures with extended conformations compared to protein-free DNA. In contrast, HMGA2 binding to linear DNA was only observed at much higher concentrations ( > 300 nM), corresponding to > 1:1 stoichiometric ratios. This caused large DNA condensates through *in cis* and *in trans* juxtaposition of DNA segments, similar to that observed for HMGA1 in an earlier study^28^.

Together, these data indicate that the close proximity of juxtaposed DNA segments within a plectonemic sc DNA molecule^29, 30^, perhaps in conjunction with DNA twist deformations, is a preferred binding substrate for HMGA2. At higher concentrations of HMGA2, protein-bound plectonemes may further interact with nearby ds DNA segments to form more compact complexes.

### HMGA2 effectively constrains plectonemic supercoils

Our results revealed that HMGA2 preferentially binds to sc DNA and induces an extended conformation of the tertiary DNA structure. We next tested whether supercoils within these complexes were constrained and refractory to relaxation by human topoisomerase I. Because (+) supercoils are formed downstream to the replication fork, the following experiments were only performed for (+) supercoils.

**Figure 4:**
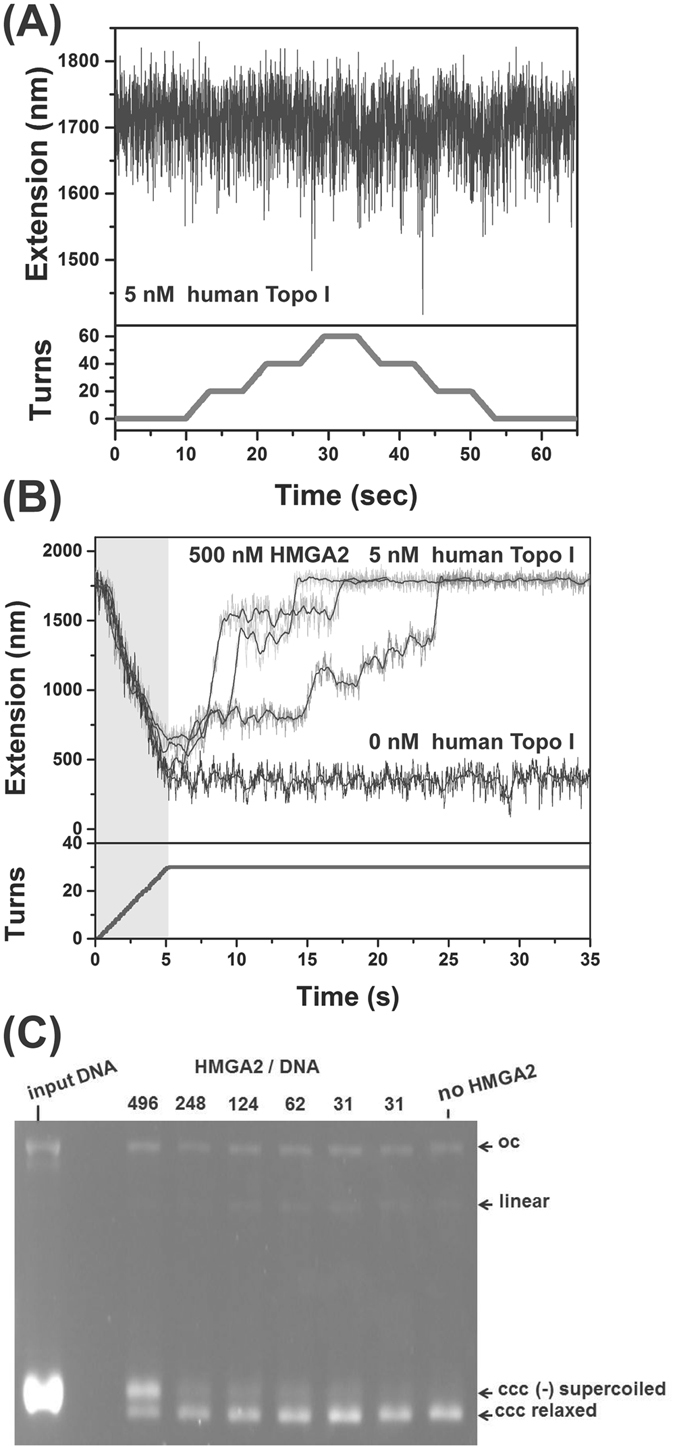
Effects of HMGA2 on supercoiling relaxation by human topoisomerase I. (**A**) A representative time-trace (top panel) of extension of a torsionally constrained DNA held at 0.3 pN during clockwise and anti-clockwise ration of the bead (bottom panel) in 5 nM topoisomerase I. The extension remains at the level of unconstrained DNA during bead rotations. (**B**) In the presence of 5 nM topoisomerase and 500 nM HMGA2, the bead rotation causes DNA supercoiling as indicated by extension decrease (gray time traces), which is slowly relaxed after the bead rotation was stopped. Data obtained in the same procedure in the absence of topoisomerase I and HMGA2 are shown for comparison (black time trace). (**C**) Analysis of covalently closed (ccc) relaxed and (−) supercoiled plasmid DNA after *in vitro* DNA relaxation reactions with human topoisomerase I. The two topological DNA forms were resolved via agarose gel electrophoresis in the presence of ethidium bromide. The stoichiometry of HMGA2 per DNA molecule in each reaction is indicated. Samples with the lowest amount of HMGA2 were run as duplicates. DNA in the left lane is 95% (−) supercoiled input plasmid DNA. See text for details and the full-length gel in Fig. S9.

The time-trace of DNA extension during rotation of the paramagnetic bead in the presence of 5 nM human topoisomerase I revealed that extension remained nearly at the level seen with relaxed DNA (Fig. 4A). Here, supercoil formation was undetectable at our recording rate of 100 Hz; therefore, the lifetime of any transiently formed supercoils must be shorter than 0.1 s. Hence, under these assay conditions, topoisomerase I prevented the formation of (+) supercoils by rapid ss DNA strand cleavage and religation, which prevents accumulation of the twisting energy of DNA. Consistent with this picture, at a 10 times lower concentration of human topoisomerase I (0.5 nM), DNA supercoil could form during rotation of the paramagnetic bead. The formed supercoil was relaxed by topoisomerase I at a single step indicated by a very fast DNA extension increase with a speed of 4.2 μm s^−1^ (Fig. S7).

In order to test whether HMGA2 constrains supercoils, we introduced a solution of 500 nM HMGA2 in the absence or presence of topoisomerase I and rotated the bead by 30 positive turns at a constant force of ~0.3 pN. In the absence of topoisomerase I and HMGA2, this corresponds to a (+) superhelical density of 0.048, and as a result, the extension dropped from ~1770 nm to ~360 nm (Fig. 4B, black data trace). In sharp contrast, in the presence of 5 nM topoisomerase I and 500 nM HMGA2, DNA extension dropped to a lesser extent after 30 rounds of bead rotations, which was gradually relaxed after the bead rotation was stopped. The relaxation process is indicated by extension increases in a jump-pause manner with irregular step sizes (Fig. 4B, gray data). Complete DNA relaxation took about 30 seconds. This is in stark contrast to the < 0.1 s supercoil lifetimes when HMGA2 was omitted from the reaction (Fig. 4A), indicating that HMGA2 can increase the supercoil lifetimes by at least two orders of magnitude. Importantly, suppression of supercoil relaxation was not observed at 100 nM where the binding to supercoiled plectonemes is saturated, as suggested by data in Fig. 3A and E. Therefore, the HMGA2 dependent constraining of supercoils at 500 nM likely requires interactions between HMGA2-bound plectonemes and ds DNA segments at the sides of the plectoneme.

We found that HMGA2 also constrained plectonemic (−) supercoils in the presence of topoisomerase I (Fig. S8). The effect of HMGA2 on constrained plectonemic (−) supercoils was also confirmed by bulk assays. Here, a fixed amount of (−) supercoiled plasmid DNA was pre-incubated with various amounts of HMGA2, and supercoil relaxation was initiated by addition of topoisomerase I. After 30 minutes, reactions were stopped and protein-free DNA analyzed through agarose gel electrophoresis in the presence of ethidium bromide. Ethidium bromide intercalated and unwound DNA, hence generating topologically overwound DNA, i.e. positive supercoiling in covalently closed (CCC) DNA. Both ccc (−) supercoiled DNA and ccc topologically relaxed DNA were converted to (+) supercoiled DNA under our experimental condition. The results showed that HMGA2 formed rather long-lived complexes with (−) sc DNA, in which the supercoils remained mostly constrained and refractory to enzymatic relaxation by topoisomerase for at least 30 minutes (Fig. 4C). Taken together, we conclude that HMGA2 stably binds to and effectively constrains both (+) and (−) plectonemic supercoils over an extended period of time.

### HMGA2 binding to supercoiled DNA requires AT-hook motifs

HMGA2 contains three AT-hooks that function as individual DNA binding domains^31^. We next tested whether multiple AT-hooks per HMGA2 molecule are required for high-affinity binding to sc DNA and employed a HMGA2 mutant that carries substitutions in AT-hooks 2 and 3 (HMGA2M), which greatly reduced DNA binding affinity^28^. The results of force-extension on linear ds DNA and of twist-extension on torsionally constrained DNA in the presence of up to 20 µM HMGA2M resulted in curves that completely overlapped with those obtained with protein-free DNA (Fig. S10). We, therefore, conclude that more than one AT-hook/HMGA2 protomer is required for high-affinity HMGA2 binding to plectonemic sc DNA.

## Discussion

We have demonstrated that the human chromatin factor HMGA2 binds with high affinity to plectonemic (+) and (−) sc DNA. These two tertiary ds DNA structures primarily differ in the orientation of their helical axis windings. Our single molecule studies, in combination with AFM, indicated that the nucleation points for HMGA2-sc DNA complex formation are located at DNA crossings where two distant segments juxtapose^32^. With more HMGA2 molecules binding, the DNA superhelix becomes covered with protein bridges. This leads to supercoil scrunching and an extended tertiary DNA structure. Our data furthermore indicated that this DNA-binding mode requires more than one AT-hook per HMGA2 molecule and constrains supercoils *in vitro*.

DNA supercoiling comes in two interchangeable geometric forms: constrained and unconstrained^33^. In eukaryotic cells, constrained supercoiling is present in nucleosomes in form of solenoidal DNA wrapping around histone octamers^34^. Unconstrained supercoiling is found in protein-free DNA and predominantly adopts a plectonemic or interwound coiling of the DNA axis^29^. Our results show that HMGA2 can kinetically suppress relaxation of plectonemic (+) and (−) sc DNA in the presence of topoisomerase I, suggesting that it has the potential to restrain unconstrained sc DNA produced downstream to the replication fork *in vivo*. The gradual, intermittent relaxation of DNA supercoiling by topoisomerase I in the presence of HMGA2 is distinct from the rapid, single-step relaxation observed in the absence of HMGA2; therefore, the observed HMGA2 dependent suppression cannot be simply explained by a reduction of accessible DNA sites for the topoisomerase I. A more likely explanation is that the HMGA2 binding to the DNA plectonemes transiently constrains the DNA supercoils into topologically isolated domains, preventing immediate complete relaxation of the DNA supercoils upon one ss DNA cleavage event by topoisomerase I. After the ss DNA cleavage is resealed, the supercoiling relaxation depends on dissociation of HMGA2 from the supercoils, result in the intermittent relaxation observed in the experiments.

Among all the DNA templates (linear ds DNA, ssDNA, forked DNA and supercoiled DNA) investigated in this study, we could detect HMGA2 binding to supercoiled DNA at the lowest concentration (~20 nM). The binding of HMGA2 to the forked DNA was detected at a concentration as low as 50 nM (Fig. 2C middle panel), suggesting the binding affinities to these two templates are comparable. Since both positively supercoiled DNA and forked DNA are generated at the replication fork, HMGA2 may play its role through a binding to both DNA supercoils and the two DNA arms at the fork, further stabilizing the (+) supercoil by bridging it to the arms and preventing the stalled replication from regression.

To our knowledge, this is the first description of a eukaryotic factor that effectively constrains both geometric forms of unconstrained sc DNA. This finding is important especially in the context of our previous HMGA2 study using human, yeast, and *E. coli* cells^11^, which showed that in all three model systems, HMGA2 prevented replication fork regression and collapse into ds DNA breaks through an association with DNA in close proximity to stalled forks. Combined with our current study, this strongly suggests that during replication stress, by transient stabilization of an HMGA2:plectoneme/daughter DNA complex, HMGA2 effectively constrains high levels of (+) supercoils that escaped relaxation by topoisomerases in the parental DNA at stalled forks. In the absence of HMGA2, the energy of (+) supercoiling can drive fork regression^16, 35, 36^, which triggers ds DNA breaks and apoptosis. This proposed role of HMGA2 in curbing unconstrained DNA supercoiling is also in agreement with our most recent discovery of functional links between HMGA2 and human topoisomerase I^37^.

It recently has become increasingly apparent that DNA topology is highly relevant in regulating transcription and replication. Transcribing RNA polymerases and translocating DNA helicases generate waves of unconstrained supercoiling, in particular when genomic DNA is immobilized through association with the nuclear matrix or by forming topologically closed DNA domains, such as proposed for telomeres^38^. Gene gating or collisions of moving replisomes with advancing RNA polymerase complexes potentially also create high levels of localized supercoiling that may escape immediate relaxation by topoisomerases and can trigger fork reversal or RNAP stalling with R-loop formation^39–41^. By combining our current and previous studies, an attractive new model implies that HMGA2 plays a global role in curbing the biological effects of unconstrained DNA supercoiling by stabilizing a DNA tertiary structure at the replication fork.

## Material and Methods

### Proteins

Human recombinant HMGA2 was purified from BL21 (DE3) Rosetta cells, following standard techniques including his-tag affinity chromatography. Recombinant human topoisomerase I was from PROSPEC.

### DNA constructs and flow-channel preparation

The detailed procedures of DNA construction including 6573 bp ds DNA (Figs 2A, 3A,B), 1192 nt ssDNA (Fig. 2B), forked DNA (Fig. 2C), and 2686 bp negative supercoiled plasmid (Fig. 3C–E) can be found in supporting information (SI: “DNA constructs”). Two types of surface modifications of flow-channels were applied according to the different labeling of DNA ends and the details were elaborated in “Flow channel preparation” in SI.

### Magnetic tweezers experiments

The magnetic tweezers setup built by our group and the force calibration method are described in our previous publication, with a relative force calibration uncertainty of ~10% due to heterogeneity in bead size^42^. A translational micromanipulator (MP-285, Sutter Instruments) was used to control the height of a pair of Neodymium magnets in order to generate constant forces. A rotation stage (DT-50, Physik Instruments) was used to wind/unwind torsion-constrained DNA tethers by rotating the magnet pair in clockwise/counter-clockwise direction, respectively.

Due to the off-center attachment that often happens in magnetic tweezers experiments, the height difference between two different forces is due to both DNA extension change and bead rotation^42^. In general, therefore, the bead height change is not equal to extension change of DNA. Only when the DNA extension change takes place at a constant force (e.g., Fig. 3A,B and Fig. 4A,B) or when DNA extension is significantly longer than the bead size (e.g., Fig. 2A), the bead height change during force change is indicative of DNA extension change (e.g., Fig. 2A where 1 μm beads were used). In the case of ss DNA manipulation experiments (Fig. 2B), the ss DNA was produced by force-induced strand-peeling transition^19, 43^ from a ds DNA. Its extension can be accurately determined using the original ds DNA tether as a reference, which eliminates the contribution from bead rotation^19, 44^. In the forked DNA experiments (Fig. 2C), the contribution from 3 μm bead rotation to bead height change cannot be ignored; therefore the bead height change during force change was directly used in the figure.

In experiments, the height of beads was tracked in real time, with ~2 nm spatial resolution for 1–3 μm beads stuck on the surface at 100 Hz. For a tethered bead, at each force, the tether was held for 30 s during which the average height was obtained, which produced the force-extension curves (Fig. 2A,B). The *Lk*-extension curve data were recorded at the force of 0.3 pN by measuring the DNA extension for 20 s at different magnet turns. The buffer solution used in the experiments contained 100 mM KCl and 10 mM Tris (pH 7.4). All experiments were conducted at 23 ± 1 °C.

### DNA tether formation

In experiments, a single DNA was tethered between a streptavidin coated 1-μm-diameter paramagnetic bead (Dynabeads MyOne) and a coverslip. In the case of stretching torsionally relaxed linear dsDNA and supercoiled DNA, the surface was functionalized with anti-digoxygenin. The dsDNA (6573 bp) was ligated with a 510 dsDNA handle containing ~50 biotinylated dUTP at one end and a 510 dsDNA handle containing ~50 digoxygenin-dUTP at the other end. For DNA that didn’t contain nicks, binding of multiple biotins to bead and multiple digoxygenins to surface imposed a constraint on the linking number of DNA, which can be changed by rotating the bead. For DNA containing one or more nicks, the DNA was torsionally relaxed. Whether a DNA is torsionally constrained or relaxed could be easily determined by rotating the bead in experiments: at forces below 0.7 pN, torsion-constraint DNA would develop supercoils, while the torsion-relaxed ones would not.

In the case of ssDNA (1192 nt) stretching, the ssDNA tether was produced from force-induced melting transition of an original dsDNA (1192 bp), which was tethered to the 2.8-μm-diameter paramagnetic bead (Dynabeads M-280) through a single biotin and to the surface through a single SH group on the same strand (see details in SI). After force-induced melting at low salt condition^45–47^, the untethered strand would dissociate into the solution. The dissociated strand would never come back since the effective concentration of free complementary strand was near zero.

### Atomic force microscopy (AFM)

The glutaraldehyde-coated mica was used for AFM imaging in order to preserve the native conformation of protein-DNA complex as described in our previous studies^25, 48^. A freshly cleaved mica was first incubated with 0.1% (3-aminopropyl)triethoxysilane (APTES) solution for 15 min, then rinsed with deionized water and dried by nitrogen gas. It was then incubated with 1% glutaraldehyde solution for 10 min and similarly rinsed with deionized water and dried by nitrogen gas. DNA concentration was 0.2 ng/μl (concentration of ~300 nM bp) and the protein concentrations were titrated to obtain different protein to DNA bp ratio. The same 6573 bp linear DNA for magnetic tweezers was used for the AFM imaging. Supercoiled DNA templates used for AFM images were pUC19 (2686 bp) plasmid.

### *In vitro* plasmid relaxation assays and topological analysis

For each sample, 300 ng of (−) supercoiled Renilla reporter plasmid (Promega) was incubated with HMGA2 for five minutes at room temperature in 50 mM Tris-Cl, pH 7.5; 100 mM KCl; 1 mM DTT; 10 mM EDTA; 5 µg/ml acetylated BSA (Life Technologies). DNA relaxation was initiated by the addition of 12 ng topoisomerase I per sample, and incubation was stopped after 30 minutes at 37 °C with 0.5% (w/v) SDS. Samples were digested with proteinase K for 20 minutes at 37 °C, and plasmid DNA purified via PCR purification kit (Qiagen). The topological state of DNA was analyzed by electrophoresis overnight in 0.8% agarose gel electrophoresis in 0.5 x TBE in the presence of ethidium bromide added to the gel. DNA was visualized under UV.

## Electronic supplementary material

Supplementary Information
