# Supplementary material for: Oncofetal HMGA2 effectively curbs unconstrained (+) and (−) DNA supercoiling
Source: Sci Rep. 2017 Aug 16;7:8440. doi: 10.1038/s41598-017-09104-5 (PMC5559611; doi:10.1038/s41598-017-09104-5)
Supplement: Supplementary file 1 — Supplementary Information [file 41598_2017_9104_MOESM1_ESM.pdf]

Supporting information

Oncofetal HMGA2 effectively curbs unconstrained (+) and (-) DNA supercoiling

Xiaodan Zhao, Sabrina Peter, Peter Dröge & Jie Yan

DNA constructs

The ds DNA (6573 bp 48% AT) was generated by PCR from the 48502 bp phage-λ DNA (New England Biolabs (NEB)) using Q5 Hot start polymerase (NEB). Two DNA handles (510 bp) labeled by multiple digoxigenin and biotin are obtained by mixing biotin-16-dUTP and digoxigenin-11-dUTP nucleotides (Roche) with dNTP solution mix in the respective PCR reaction. All three DNA pieces were ligated by incubating them with T4 ligase (NEB) overnight at 16°C. The above process generated two populations of DNA tethers, i.e. the torsionally constrained (nick-free) DNA and torsionally unconstrained DNA, which is due to the nick incurrence during the sample preparation. Therefore, the torsionally unconstrained DNA was used in HMGA2 binding to linear ds DNA and the torsionally constrained DNA was used in HMGA2 binding to DNA supercoiled plectonemes.

The 1192 nt ss DNA tethers were obtained from initially ds DNA (1192 bp, 54% AT) tethers that were produced by PCR and labeled by 5'-biotin and 3'-thiol on the same strand. The individual ds DNAs were tethered by M280 paramagnetic beads (Invitrogen) which can apply force range from 0.1 pN to > 100 pN. Through force-induced DNA strand-peeling transition at ~ 65 pN under low ionic strength[1-3], an individual ss DNA tether can be generated by dissociating the un-tethered ss DNA strand[4]. The un-tethered ss DNA diffuses away and has no chance to rebind on the tethered ss DNA.

The fork DNA was constructed by ligating a hairpin with two ds DNA handles. The hairpin was composed of 52 bp hairpin stems of non-consensus sequences (50% AT) and an eight nt poly-A stem loop, which was directly purchased from Integrated DNA Technologies (IDT). Two DNA handles (497 bp and 672 bp) were both labeled on 5' end with biotin and thiol, respectively.

In order to obtain pure negative sc DNA plasmids, Puc19 (2686 bp) plasmids purchased from NEB were transformed in DH5 alpha cells. It follows by the miniprep purification (QIAprep) and gel extraction to remove the small amount of linear and circular DNA.

Flow channel preparation

The flow channel was first made from two #1 glass coverslips and the bottom one was functionalized by 3-Aminopropyl)triethoxy silane (APTES). The channel was further modified depending on the labeling of DNA end. For the digoxigenin labeled DNA constructs, the channel was then flushed by Silane-PEG-NHS (PG2-NSSL-5k, Nanocs) dissolved in DMSO and incubated for 1 hour. It was followed by flushing anti-digoxigenin Fab fragments (Roche) in the channel to create covalent bonds with NHS group on PEG and incubated for another 1 hour. Lastly filling the channel with 1% BSA solution in 1× phosphate buffered saline (PBS) buffer (pH 7.4) and incubated overnight at 4°C before experiments to avoid non-specific binding of DNA to the surface.

For the thiol labeled DNA constructs, the channel was then flushed by sulfo-SMCC (Thermo Scientific) dissolved in 1xPBS buffer (pH 7.4) and incubated for 30 min. After rinsing out unbound sulfo-SMCC, thiol-labeled DNA constructs were introduced into the chamber and

incubated for another 30 min. Finally, the channel was filled by 1% BSA solution in 1xPBS buffer (pH 7.4).

Supplimentary figures

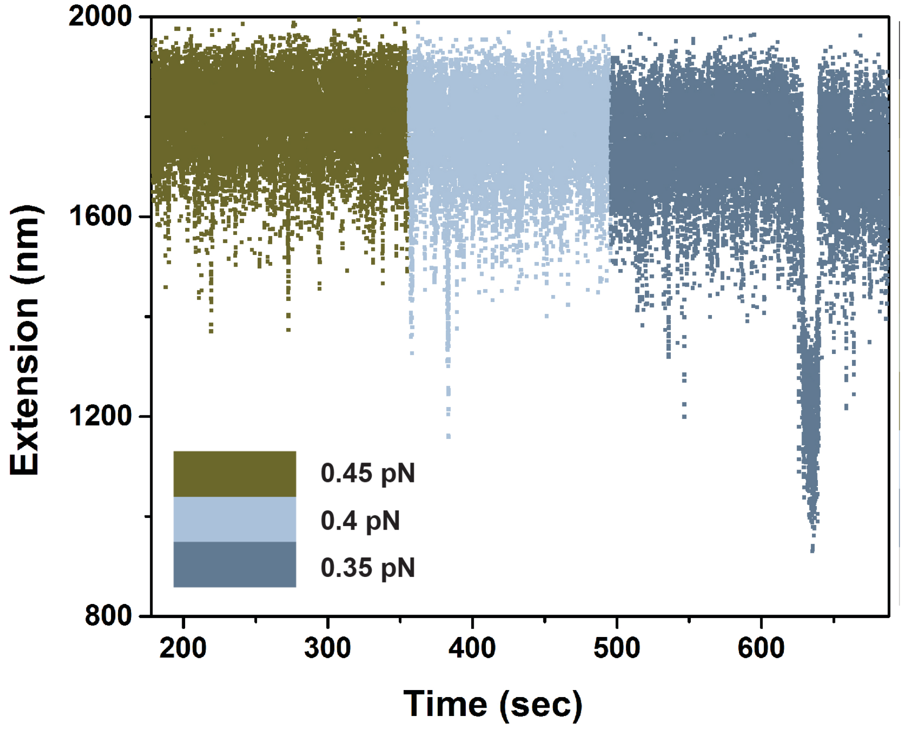

Figure S1. In the presence of 1000 nM HMGA2, large amplitude extension fluctuation of DNA was observed at small forces from 0.35 to 0.45 pN, indicating that the DNA condensation is through highly dynamic weak interactions mediated by HMGA2.

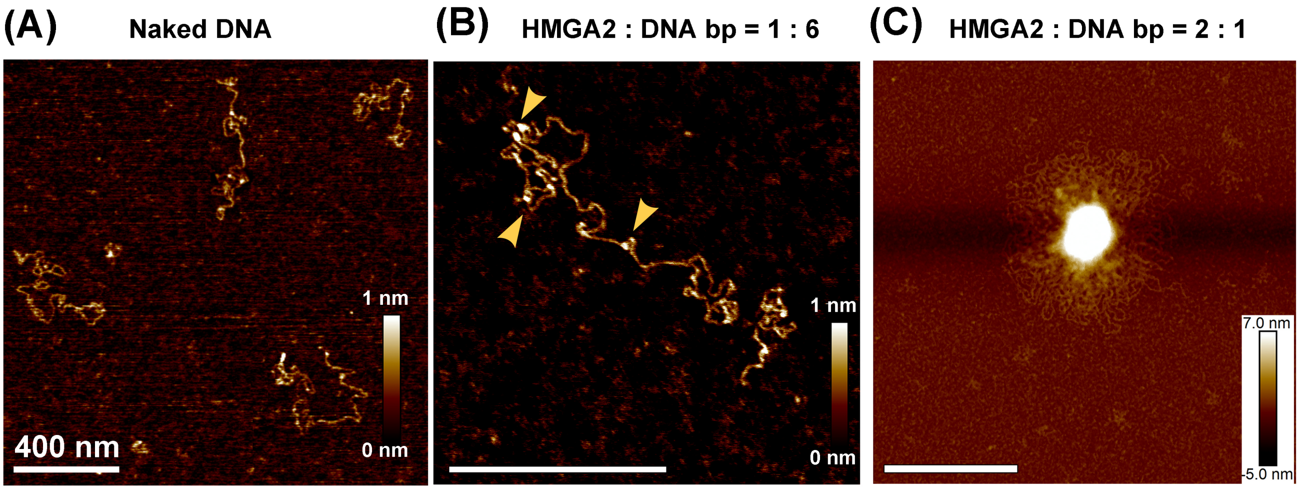

Figure S2. AFM images of 6.5 kbp linear DNA (B-C) bound with HMGA2 with different HMGA2 : DNA base pair stoichiometric ratios. Images obtained on naked linear DNA (A) is shown for comparison.

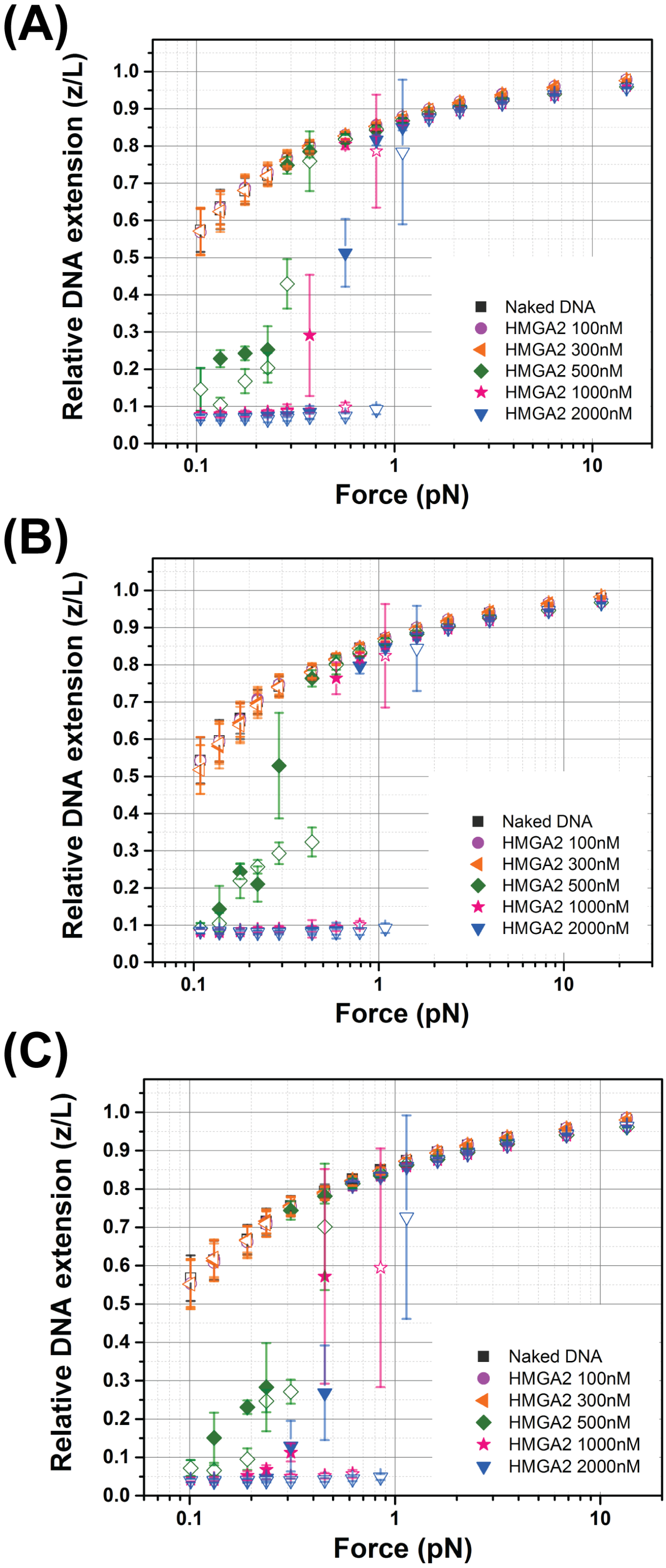

Figure S3. Three representative independent experiments, in addition to Figure 2A described in the main text, to demonstrate the reproducibility of the effects of HMGA2 on the force-extension of torsionally unconstrained DNA.

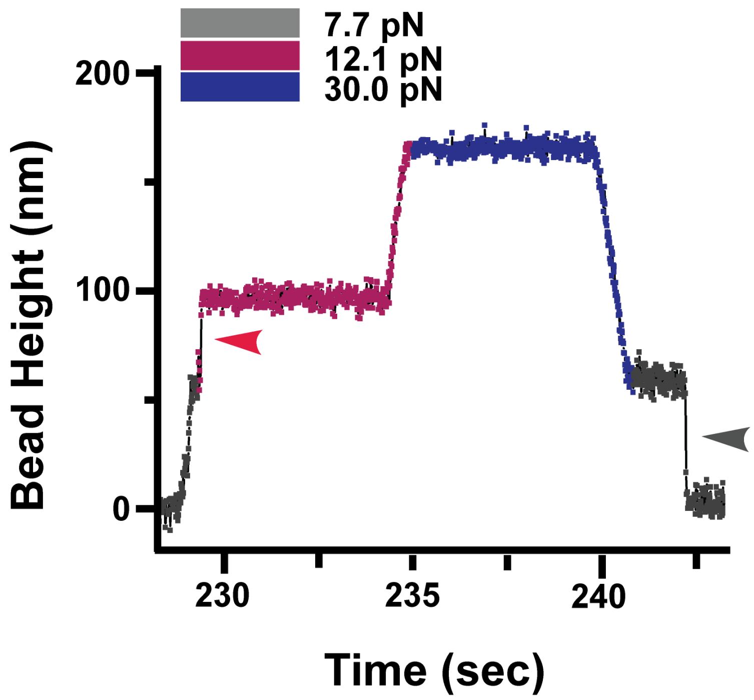

Figure S4. A representative force cycle (7.7±0.8 pN → 12.1 ±1.2 pN → 30.0±3.0 pN → 7.7±0.8 pN) shows unfolding of a naked DNA fork at 12.1 pN (indicated by red arrow) and refolding at 7.7 pN (indicated by gray arrow) events. This cycle is extracted from the last cycle in Fig. 2C, top panel.

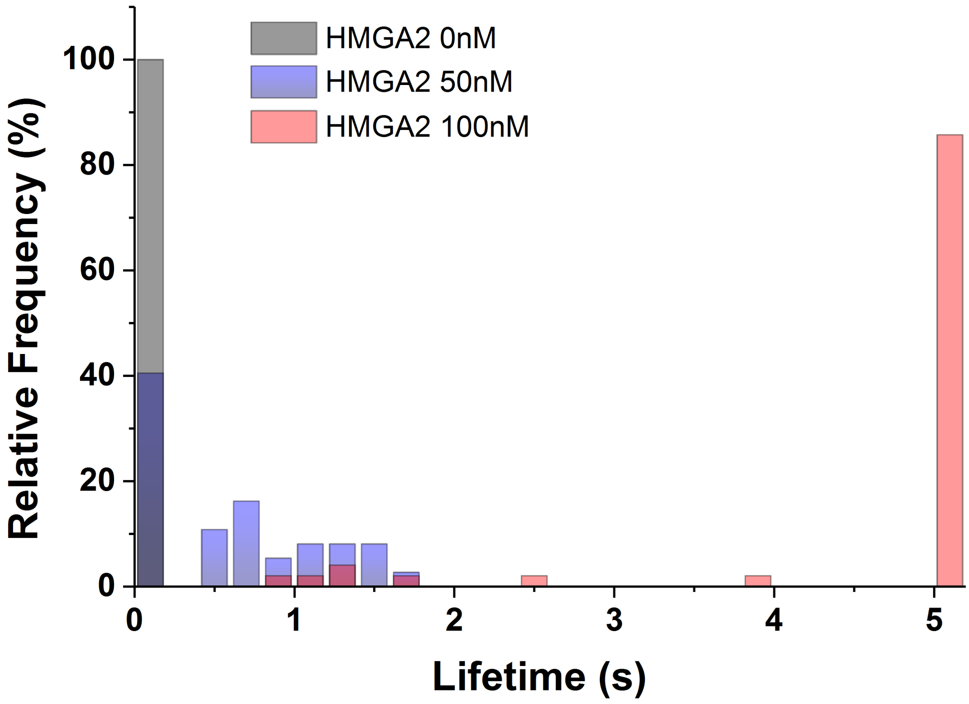

Figure S5. Related to Figure 2C. Lifetime histogram of hairpin in the folded state at F_l in the presence of varied HMGA2 concentration. If the lifetime is below 0.1 second, the valude was rounded to 0.

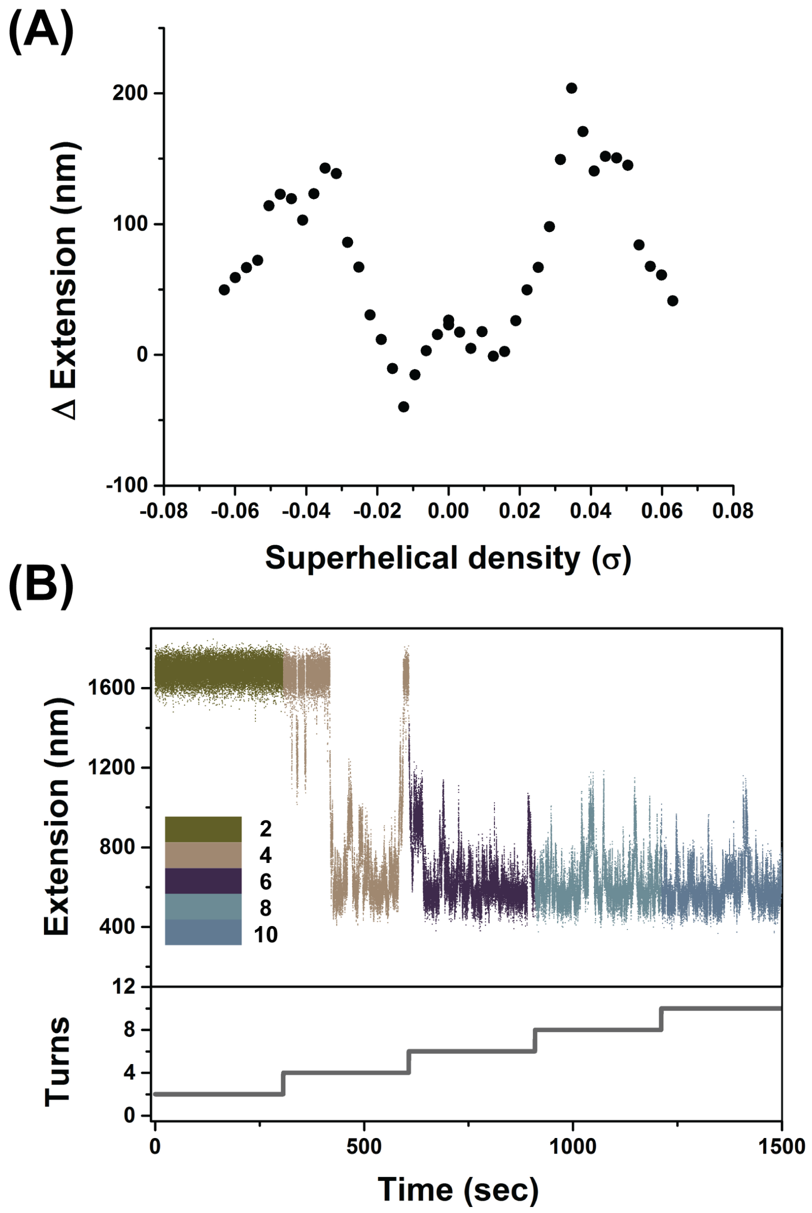

Figure S6. (A) Extension difference of the *Lk*-extension curves of a torsionally constrained DNA held at 0.3 pN between 100 nM HMGA2 and naked DNA (**Fig. 3A**). (B) The large amplitude of extension fluctuation at constant supercoiling densities for 300 sec in 500 nM HMGA2, indicating a highly dynamic DNA condensation mediated by HMGA2.

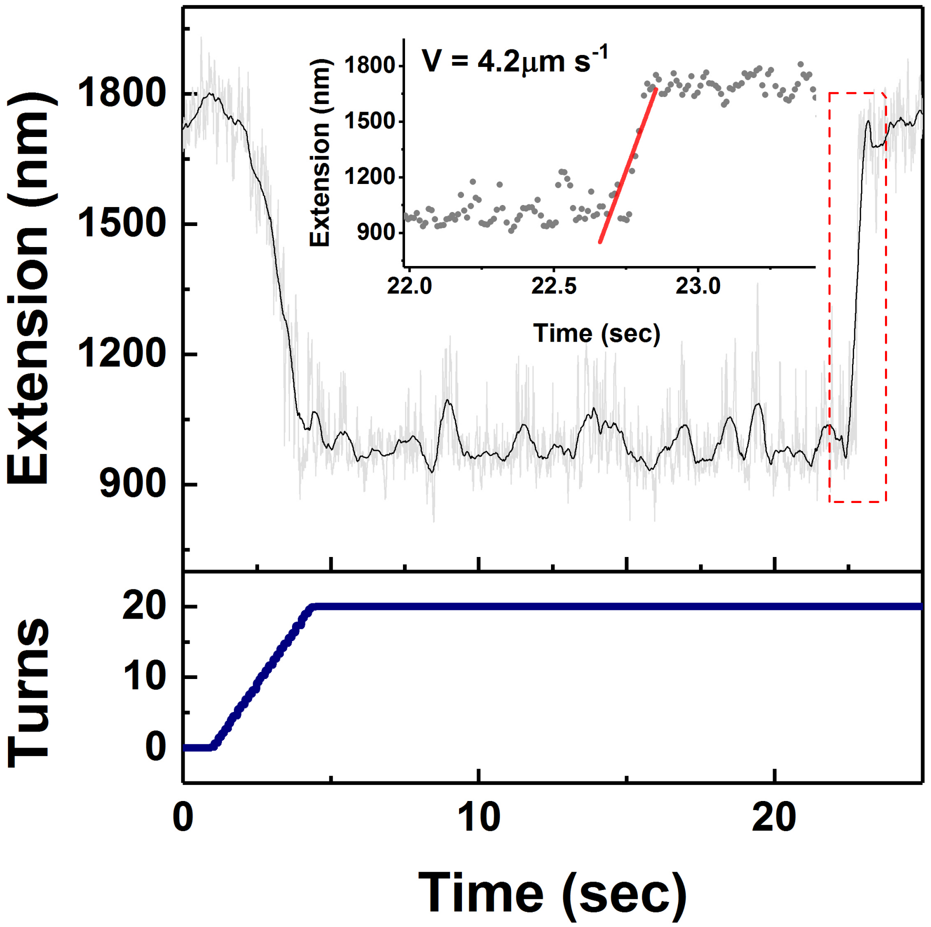

Figure S7. The time-trace of DNA extension during rotation of the paramagnetic bead in the presence of 0.5 nM human topoisomerase I. The reduction of DNA extension upon the bead rotating indicates the formation of supercoiled DNA. A single step in DNA extension increase suggests a single supercoil removal event by topoisomerase I with an extension increase speed of 4.2 μm s^{-1} (Insert).

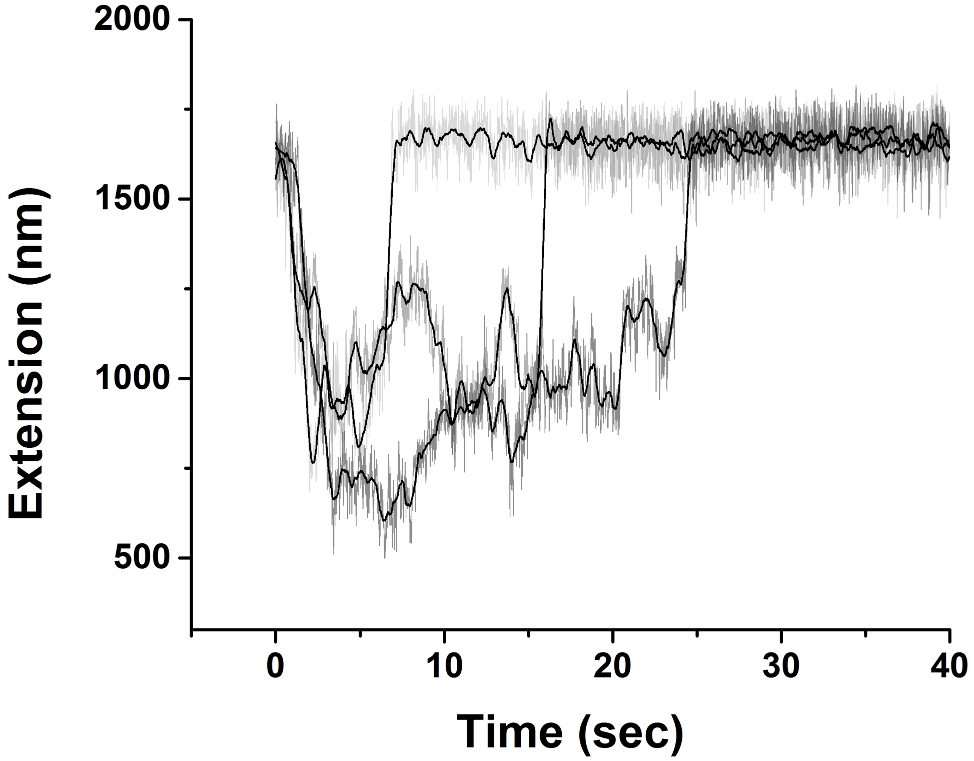

Figure S8. Effects of HMGA2 on the relaxation of negatively supercoiling DNA plectonemes by human topoisomerase I. Three representative time traces of extension obtained from a torsionally constrained DNA held at 0.3 pN in the presence of 5 nM topoisomerase I.

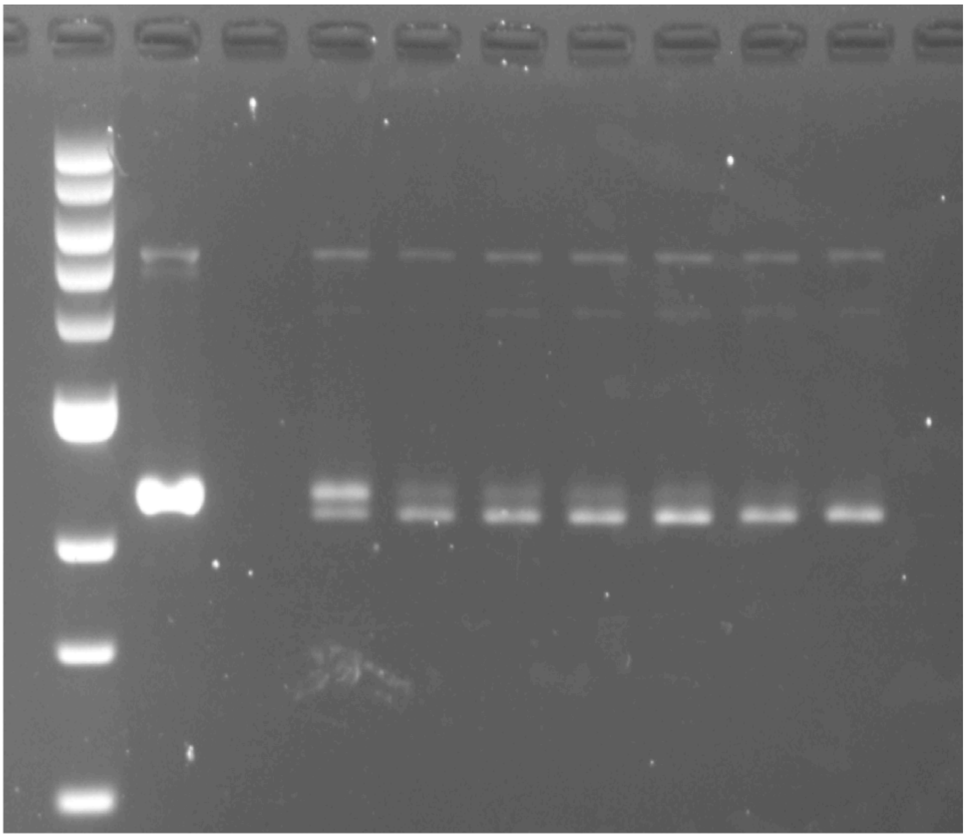

Figure S9. Related to Figure 4C. The full-length gel of analysis of covalently closed (ccc) relaxed and (-) supercoiled plasmid DNA after in vitro DNA relaxation reactions with human topoisomerase I. The ladder is 1kb marker from NEB.

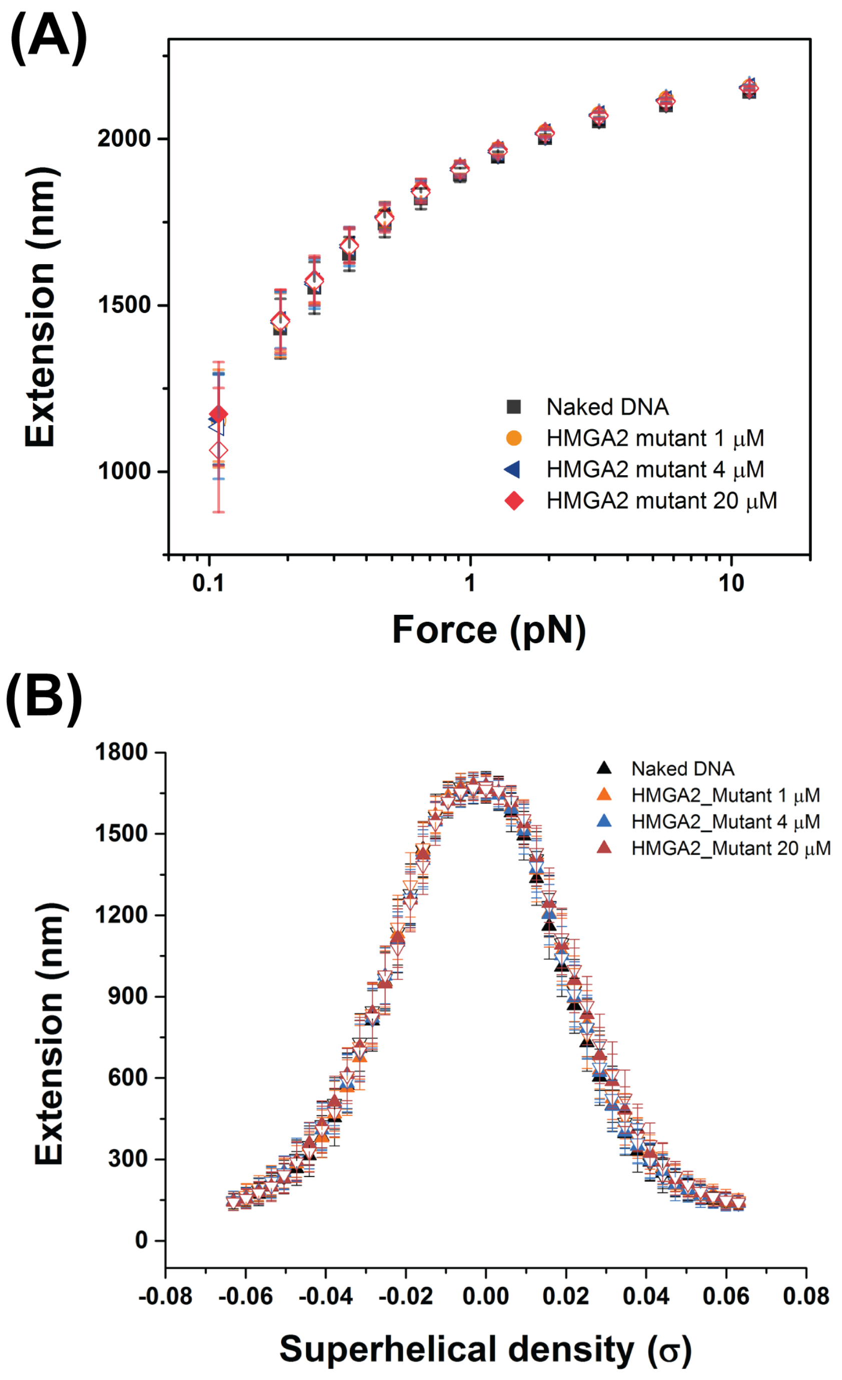

Figure S10. Binding of HMGA2M to linear ds DNA and sc DNA. FEC of linear ds DNA (A) and *Lk*-extension of sc DNA (B) results show no apparent binding for HMGA2M of concentration up to 20 μM.

Reference

1 Fu, H. *et al.* Transition dynamics and selection of the distinct S-DNA and strand unpeeling modes of double helix overstretching. *Nucleic acids research* **39**, 3473-3481, (2010).

2 Zhang, X., Chen, H., Fu, H., Doyle, P. S. & Yan, J. Two distinct overstretched DNA structures revealed by single-molecule thermodynamics measurements. *Proceedings of the National Academy of Sciences of the United States of America* **109**, 8103-8108, (2012).

3 Zhang, X. *et al.* Revealing the competition between peeled ssDNA, melting bubbles, and S-DNA during DNA overstretching by single-molecule calorimetry. *Proceedings of the National Academy of Sciences* **110**, 3865-3870, (2013).

4 Fu, H., Le, S., Chen, H., Muniyappa, K. & Yan, J. Force and ATP hydrolysis dependent regulation of RecA nucleoprotein filament by single-stranded DNA binding protein. *Nucleic acids research*, (2012).
